# Epidemiological, Serological, and Entomological Investigation of New Visceral Leishmaniasis Foci in Nepal

**DOI:** 10.4269/ajtmh.23-0373

**Published:** 2023-11-27

**Authors:** Anand Ballabh Joshi, Megha Raj Banjara, Murari Lal Das, Pragyan Ghale, Krishna Raj Pant, Uttam Raj Pyakurel, Gokarna Dahal, Krishna Prasad Paudel, Chuman Lal Das, Axel Kroeger, Abraham Aseffa

**Affiliations:** ^1^ Public Health and Infectious Disease Research Center (PHIDReC), Kathmandu, Nepal;; ^2^ Central Department of Microbiology, Tribhuvan University, Kirtipur, Kathmandu, Nepal;; ^3^ UNICEF/UNDP/World Bank/WHO Special Programme for Research and Training in Tropical Diseases (TDR), Geneva, Switzerland;; ^4^ Epidemiology and Disease Control Division, Department of Health Services, Teku, Kathmandu, Nepal;; ^5^ Centre for Medicine and Society, Albert-Ludwigs-University, Freiburg, Germany

## Abstract

The aim of this study was to explore epidemiological, serological, entomological, and social aspects of visceral leishmaniasis (VL) in new foci in Nepal. The study was conducted in 11 villages of five districts that had been previously free of VL but that reported new cases between 2019 and 2021. We screened 1,288 inhabitants using rK39 tests and investigated the epidemiological and clinical characteristics of 12 recent VL cases. A total of 182 community members were interviewed about knowledge, attitude, and practices regarding VL. They then underwent an awareness training; 40 of them had a second interview at 6 months to assess the training impact. Vector surveys were conducted in six houses per village to assess sandfly density and infection rates. The prevalence of VL infection was 0.5% and 3.2% among screened populations in Dolpa and Kavre districts, respectively, while the other districts had no rK39-positive cases. No association between travel history and VL infection was found. *Phlebotomus argentipes* sandflies were collected in three districts at high altitudes (from 1,084 to 4,450 m). None of the sandflies captured had *Leishmania donovani* DNA. People in new foci were not aware of VL symptoms, vectors, or preventive measures. The training significantly improved their knowledge and practice in seeking medical care in case of illness. The epidemiological, serological, and entomological investigations suggest indigenous focal transmission of VL. An integrated package of strategic interventions should be implemented by the national VL elimination program in districts with new VL foci.

## INTRODUCTION

Visceral leishmaniasis (VL) or kala-azar is a fatal vector-borne disease caused by *Leishmania donovani* and transmitted on the Indian subcontinent by the sandfly *Phlebotomus argentipes*. The WHO Road Map for Neglected Tropical Diseases 2021–2030 has a target of 32 countries validated for elimination of VL as a public health problem (defined as < 1% case fatality rate due to primary visceral leishmaniasis) by 2023, 56 by 2025, and 64 by 2030.^1^ Bangladesh, Nepal, and India are close to VL elimination, with the achievement of less than 1 infection per 10,000 population each at the subdistrict, district, and block levels. ^2^ In recent years, transmission of VL in new foci previously nonendemic for VL and *de novo* transmission of VL in areas where it had been stopped over many years have emerged as a significant challenge to VL elimination in the Indian subcontinent.

The nationwide proportion of VL cases from new foci has been on the rise in Nepal in recent years, constituting 33% of all VL cases in 2017, 49% in 2018, and 61% in 2019.^3^ Although the proportion of VL cases from new foci has been increasing, there is no program of investigation and control of VL in these areas.

Visceral leishmaniasis cases have been reported in Nepal from 50 of its 77 districts. In 2017, 65% of reported VL cases in Nepal were from 31 districts where VL was considered non-endemic.^4^ The national guideline on kala-azar elimination defines the district as being endemic for kala-azar if there is a full cycle of transmission at any given time, including a competent vector and a parasite reservoir, and at least one indigenous case in the last 10 years. The district is non-endemic for kala-azar if it has no full cycle of transmission and no locally acquired case has been reported in the last 10 years.^4^ Sporadic cases have been consistently reported from different parts of the country, including hilly districts. Routine reports from the national VL elimination program suggested that there could be indigenous transmission of VL. However, no systematic studies, including epidemiological, serological, and entomological investigations, of VL have been conducted in these suspected new foci so far.

The transmission dynamics of VL in these new foci and the drivers of this transmission are largely unknown. The role of the *P. argentipes* sandfly vector in the transmission of VL to humans has been well established in the Southeast Asia region in Bangladesh, India, and Nepal.^5^ Since no effective vaccine is available for the prevention of VL, the control of transmission depends on the reduction of sandfly vector densities, mostly of *P. argentipes*. Although information about breeding places and the seasonal variation of vector densities in areas endemic for VL has been available in the Indian subcontinent,^6^ such information is lacking from new foci. A recent study of new foci of two districts detected a very low density of sandflies.^7^ An understanding of sandfly vector species, ecology and behavior of local vectors, and environmental aspects and an adequate understanding of the epidemiology of VL are essential for effective vector control and disease prevention approaches and would ensure the use of appropriate evidence-based vector control interventions using vector surveillance information in support of the regional VL elimination initiatives.

Knowledge, attitude, and practices (KAP) in relation to sandflies and VL have not yet been investigated in new focal areas of VL in Nepal. This survey documented baseline KAP of the communities across the selected study districts among a range of age groups, sex, and social classes. The main aim of this study was to explore epidemiological, serological, and entomological aspects of VL in suspected new VL foci and to assess the knowledge, attitude, and practice towards VL of people living in these areas.

## MATERIALS AND METHODS

### Ethical issues.

This study received ethical approval from the WHO Ethics Review Committee (reference number ERC 0003530) and the Nepal Health Research Council in Nepal (reference number 3083). In this research, an investigation was conducted into suspected new VL foci, including early detection of VL and post-kala-azar dermal leishmaniasis (PKDL) cases by index-based active case detection (i.e., screening for secondary cases in villages with a recently detected VL case) and sandfly density measurement by the research team in coordination with the National Kala-Azar Elimination Program. For the epidemiological investigation of VL cases, and serological investigation of household members, the research team conducted observational studies and interviewed VL case and household heads. Blood samples were collected from household members for rK39 tests after their consent and assent were obtained where applicable. The research team also conducted entomological studies in and around households reporting VL cases. For these purposes, voluntary written informed consent was obtained.

### Study design.

This study investigated the transmission of VL in new foci districts through epidemiological, serological, and entomological investigations. New foci were defined as areas where VL transmission had not been reported for at least the last 10 years but where at least one new VL case has been recently reported.^4^ Based on reports of previously confirmed VL from new foci in 2019 and 2020, and recently confirmed VL cases in 2021, the villages with VL cases were selected for the study. Vector abundance was surveyed in households with reported VL cases and surrounding households in the village. Serological tests of inhabitants of those villages were performed with the rK39 test, and a detailed epidemiological investigation was carried out for recent or previously confirmed VL cases.

### Study sites and population.

**Figure 1.**
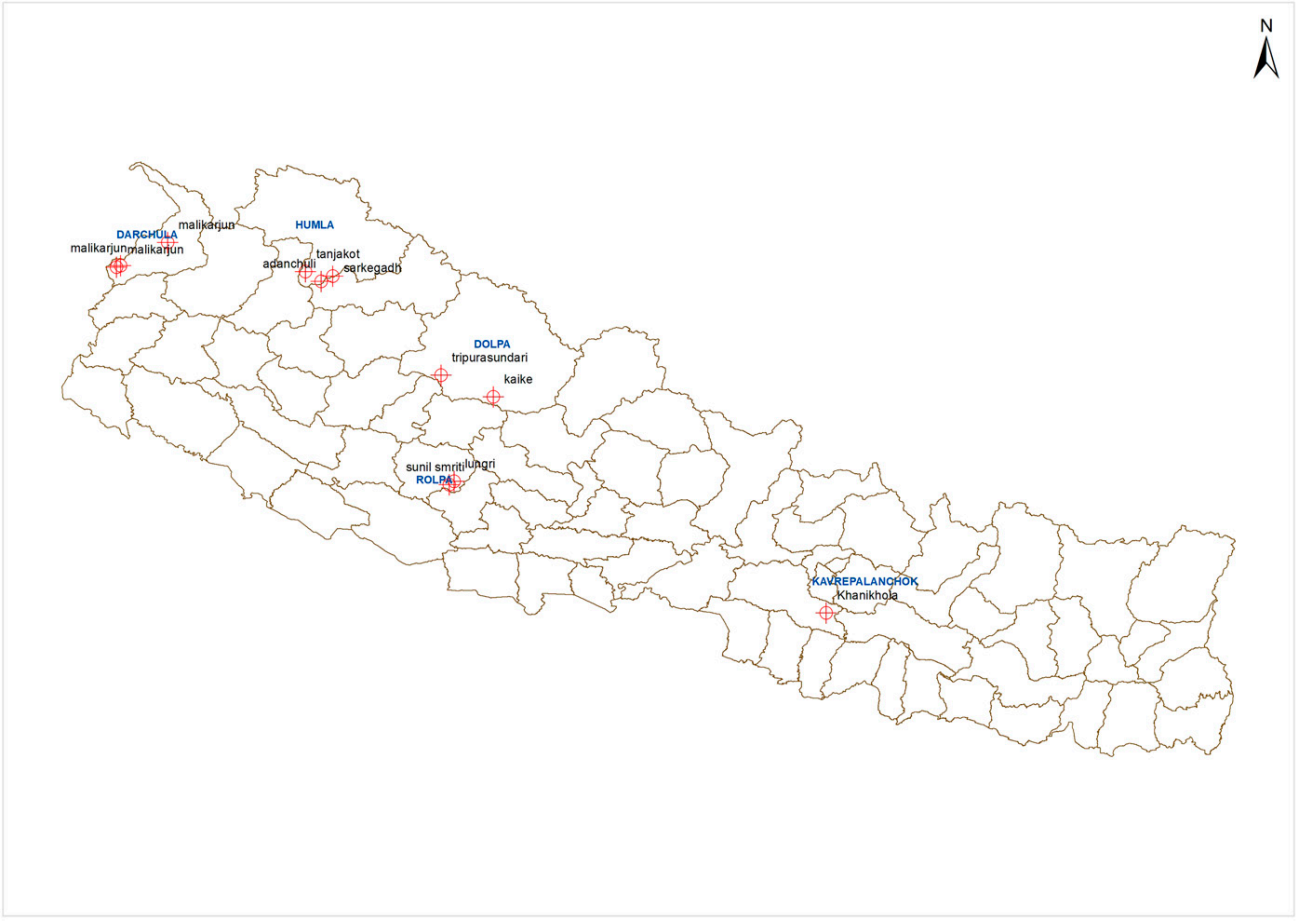
Location of study sites.

The investigation of suspected new VL foci was conducted from July 2021 to September 2022 in five districts, namely, Kavre, Rolpa, Dolpa, Humla, and Darchula, reporting VL cases within the last 3 years (as described below; Figure 1).

In suspected new VL foci, the household of a VL case and the households within a 500-m radius were investigated for VL. Upon informed consent, blood samples were collected from all inhabitants above 2 years of age for serological testing. A detailed epidemiological investigation (as described below) was carried out on those individuals who were confirmed VL cases as well as those who were found positive by the serological test but were asymptomatic. Further, vector density was measured in six to eight households in and around the household with a reported VL case using CDC light traps and manual aspiration.

### Sample size and sampling.

All recently confirmed VL cases (i.e., VL cases reported within the last 3 years through the national program) in five suspected new foci were investigated.

For serological investigation, including asymptomatic infection, we collected blood samples from all inhabitants above 2 years of age residing in villages of suspected new VL cases and tested them using the rK39 test. For the rK39 test, the geographical boundary of sampling was set at a 500-m radius of a household with a VL case.

For entomological investigations, VL-positive households and the surrounding six to eight households were included in the collection of sandflies using CDC light traps over two consecutive nights. In addition, sandflies were collected manually in the same households using aspirators.

### Coordination with national VL program and community engagement.

The suspected new foci for investigation were identified in coordination with the Epidemiology and Disease Control Division (EDCD) in Nepal. Program staff from national, provincial, and district levels were involved in the investigation of suspected new foci. The researchers from the Public Health and Infectious Disease Research Center (PHIDReC) and staff from the EDCD jointly performed verification of occurrence of VL cases, epidemiological and clinical investigations, serological tests for detection of asymptomatic infections, measurement of sandfly density, and assessment of awareness of members in the community. Training sessions in the communities were carried out by the District Health Office.

The EDCD provided administrative support to the research team to advance the project activities as per its objectives and to identify suspected new foci and VL patients. They ensured coordination with the Ministry of Health at the province level and supported building a strong linkage between partners, including province-, district-, and municipality-level health systems.

Community meetings were conducted at local health posts, and female community health volunteers (FCHVs), community leaders, schoolteachers, and staff members of local health facilities were invited prior to the community survey. They were informed about VL joint project activities and were asked to support the investigation. The community was made aware of VL transmission risk and its prevention so that the community stigma could be minimized. Separate attempts were made to connect the schoolteachers, FCHVs, and community leaders to achieve a positive attitude toward VL investigation.

### Data collection procedure.

*Verification of occurrence of VL.* The number of VL patients reported from suspected new foci from 2019 to 2021 was obtained from the District Health Office and the EDCD, Nepal. The villages were visited, and the VL cases were identified. The case verification was done only upon written informed consent. The patient or the household head (in the case of minors) was asked about the occurrence of the disease, date of diagnosis, place of diagnosis, treatment received, and treatment outcome. If the hospital treatment card was available during the interview, it was analyzed.

#### Epidemiological and clinical investigations.

Once the case of VL was verified, further epidemiological and clinical characteristics were explored. Demographic characteristics, including age, sex, household size, occupation, and education level of the VL patients, were collected. Information on house characteristics and the nearby external environment favorable for sandfly breeding and geographical data were collected by direct observation using an observational checklist. Clinical data, including signs and symptoms of VL, duration of illness, any biochemical parameters if monitored during the illness of the VL patient, and any consequences if a hospital treatment card was available, were recorded. Travel history of the VL-affected person within 1 year and other VL-related risk factors were asked about and recorded.

#### Serological tests for detection of asymptomatic infections.

In a village with a confirmed VL case, blood samples of all residents above 2 years of age of that village were collected to detect any asymptomatic infection. Fifty to 200 households, depending upon household numbers in the villages, were covered for screening of VL. The average village sizes in Nepal are between 50 households (hilly districts) and 150 households (Terai districts). rK39 tests (Kalazar Detect^TM^ rapid test; InBios International USA) were performed at the household using the blood samples, and results were recorded. Written informed consent was obtained from each participant before collection of finger prick blood samples for the rK39 test. rK39 test-positive individuals were referred to the hospital for confirmatory VL diagnosis and treatment.

#### Measurement of sandfly density, identification, and parasite positivity rate in collected sandflies.

Vectors were collected using CDC light traps for two consecutive nights in a VL case household and additionally in six to eight houses around the VL case household. Vectors were collected by trained personnel under the guidance of an entomologist from the study team. Light traps were placed in a corner of the house from 6 pm to 6 am, 2 inches away from the wall, with a distance of 6 inches between the floor and the bottom of the sac. Sandflies were also collected manually using an aspirator. Sandflies were separated as males and females, and species were identified under the microscope. Sandfly density was calculated as the mean number of sandflies per CDC light trap per night. Morphological identification of sandflies was done in the field. Collected sandflies were preserved in 80% ethanol.

Parasite DNA was extracted from collected *P. argentipes* and other sandflies (QIAamp^®^ DNA mini kit, Qiagen, Hilden, Germany; lot number 51304). kinetoplast DNA (kDNA) of *L. donovani* was amplified using a species-specific polymerase chain reaction (PCR) (ProFlex^TM^ thermocycler; Applied Biosystems, Waltham, MA).^8^ The PCR product was applied in 1% agarose gel.

#### Assessment of awareness of VL of community members in new VL foci and training sessions.

Community members were interviewed using structured questionnaires regarding their awareness of visceral leishmaniasis as a disease and its transmission, the vector, prevention methods, their treatment-seeking behavior, and their attitude towards the disease.

Health workers from the local health post and the research team conducted awareness sessions, including information, education, and communication about VL, after the KAP survey. Community members, with at least one person from each household, preferably the household head, were asked to gather in a public place (such as the school or health facility of the village) to receive information on VL.

### Data management and analysis.

A well-checked data entry program was designed using the Kobo Toolbox survey application from the United Nations Office for the Coordination of Humanitarian Affairs (UNOCHA). Data quality was maintained as per data management guidelines. Descriptive statistics were generated.

## RESULTS

### Serological investigation for VL in suspected new foci.

Clinical and serological investigations were conducted in Kavre, Humla, Dolpa, Rolpa, and Darchula. Of 1,288 individuals screened using rK39, 668 (51.8%) were female.

Three participants from Dolpa and Humla had suffered from fever 2 weeks prior to the survey. No participants had skin lesions suggestive of PKDL. There were three participants from Dolpa who had spleen enlargement. Four participants had a positive rK39 test (two from Dolpa district with fever and splenomegaly and two from Kavre district without fever or splenomegaly). The symptomatic/asymptomatic both VL infections at the time of the survey were 0.5% and 3.2% among the screened populations in Dolpa and Kavre, respectively. The three other districts, namely, Darchula, Rolpa, and Humla, had no rK39-positive cases among the screened population (Table 1).

**Table 1:** VL-related information among participants of serological survey

Particulars	Darchula	Rolpa	Dolpa	Humla	Kavre
Number of population screened	75	181	371	598	63
Number of villages covered	3	2	2	3	1
Number of participants with:					
Fever	0	0	2	1	0
PKDL-like skin lesions	0	0	0	0	0
Enlarged spleen	0	0	3	0	0
rK39 positivity	0	0	2	0	2
Percent of both symptomatic or asymptomatic infection among screened population	0	0	0.54	0	3.2

PKDL = post-kala-azar dermal leishmaniasis; VL = visceral leishmaniasis.

### Epidemiological and clinical characteristics of VL cases in suspected new foci.

New foci were selected for investigation based on the location of new VL patients reported between 2019 and 2021. Three previous VL cases in Darchula, three in Dolpa, three in Humla, and one in Kavre were investigated for their clinical characteristics. In Rolpa, there were two cases reported from 2019 to 2021; however, during the visit for investigation, one patient had died and the other was out of the country. Similarly, one previous VL case in Humla had already died at the time of investigation (Table 2).

**Table 2:** Clinical characteristics of VL patients in new foci*

Characteristics	Darchula (*N* = 3)	Dolpa (*N* = 3)	Humla (*N* = 4)	Kavre (*N* = 1)
Place of diagnosis				
Government hospital	3	2	3	1
Private hospital	0	1	0	0
Drugs used for treatment				
Single-dose liposomal amphotericin B	2	2	2	1
Miltefosine	1	1	1	0
Status of patients				
Alive	3	3	3	1
Dead	0	0	1	0
Travel history 1 year prior to VL diagnosis				
Yes	1	1	1	0
No	2	2	2	1
Patients required further treatment after treatment and discharge				
Yes	0	0	0	0
No	3	3	3	1

VL = visceral leishmaniasis.

* In Rolpa, there were reports of two VL cases, but during the investigation, one was found to have died and one was out of the country. Therefore, we could not collect the patient information for two VL cases in Rolpa.

All VL patients were diagnosed in the government hospital except for one from Dolpa, who was diagnosed in a private hospital. The majority of cases received single-dose liposomal amphotericin B, whereas a few were treated with miltefosine. All of the VL cases in Darchula, Dolpa, Humla, and Kavre were alive at the time of investigation. Three VL cases, one each from Darchula, Dolpa, and Humla, had a travel history before suffering from VL, whereas others had no history of travel. None of the VL cases had required any further treatment after treatment completion and discharge (Table 2).

The majority of the previous VL patients in new foci were male. They lived in houses made of mud and stone (12 out of 12), with visible moisture (7 out of 12), with cracks in the house wall (8 out of 12), with animal sheds under same roof (6 out of 12), with muddy surroundings (9 out of 12), and with potential sandfly breeding areas (12 out of 12) (Table 3).

**Table 3:** Epidemiological characteristics of VL patients in new foci

Characteristics	Darchula (*N* = 3)	Rolpa (*N* = 2)	Dolpa (*N* = 3)	Humla (*N* = 3)	Kavre (*N* = 1)
Age group (years)					
< 15	0	1	2	2	0
15–60	2	1	1	1	1
> 60	1	0	0	0	0
Sex					
Male	2	2	2	3	1
Female	1	0	1	0	0
Profession					
Own agriculture	1	0	0	1	1
Tailor	1	0	0	0	0
Skilled worker	1	1	1	0	0
Student	0	1	0	1	0
Others (small child)	0	0	2	1	0
Type of houses					
Mud	0	2	2	0	1
Stone and mud	2	0	0	3	0
Cement plaster	1	0	0	0	0
Wood and stone	0	0	1	0	0
Floor type					
Mud	3	2	2	3	1
Areas with visible moisture					
Yes	0	2	1	3	1
No	3	0	2	0	0
Cracks in the house					
Yes	1	2	1	3	1
No	2	0	2	0	0
Stories of the house					
One	1	1	1	1	1
Two or more	2	1	2	2	0
Animal shed locations					
Under same roof	2	1	1	2	0
Outdoor	1	1	2	1	1
Water source distance from the house					
< 100 m	3	1	3	2	0
> 100 m	0	1	0	1	1
Location of the toilet					
Inside the house	1	0	0	1	1
Outside the house	2	2	2	2	0
None	0	0	1	0	0
Characteristics of surroundings					
Muddy	1	2	2	3	1
Rocky	2	0	1	0	0
Sandfly breeding areas in house					
Yes	3	2	3	3	1
No	0	0	0	0	0

VL = visceral leishmaniasis.

### Spatial distribution of vectors and their infection rate.

The villages included in the investigation were located at 1,084 to 4,450 m altitude. The two nights of entomological monitoring was carried out in 72 households in the five districts.

Eight female *P. argentipes* sandflies were collected in Sanodhaple, Kavre district; all of them were unfed. There were no fed and gravid female sandflies in the households of Sanodhaple, Kavre. No sandflies were detected in Sanonamja village of Rolpa. In Tebang village of Rolpa, three *P. argentipes* sandflies were collected, but *Phlebotomus papatasi* and *Sergentomyia* species sandflies were not detected. A large number of *P. argentipes* sandflies was collected in Palang village of Dolpa district. Seventeen unfed, 65 fed, and two gravid *P. argentipes* sandflies were collected. There were no *P. papatasi* or *Sergentomyia* species sandflies collected in Palang, Dolpa. In Sahartara village of Dolpa district, seven sandflies were collected, and all of them were *P. argentipes*. Among them, three were males, two were unfed females, one was a fed female, and one was a gravid female. Large numbers of sandflies of all species were collected in Tallokalkhe village of Humla. Of *322 P. argentipes* sandflies, 54 were unfed females, 57 were fed females, and nine were gravid females. In comparison, the density of other species was lower. In Tumcha village of Humla district, 23 female *P. argentipes* and 1 female *P. papatasi* sandflies were collected. No sandflies were collected from Madana village of Humla district. Both *P. argentipes* and *P. papatasi* sandflies were collected in Firudi village of Darchula. Two fed *P. argentipes* females were collected in Firudi, Darchula. In Khuna village of Darchula district, *15 P. argentipes* females and *20 P. papatasi* females were collected. Among the *P. argentipes* and *P. papatasi* sandflies collected in Lethya village of Darchula, eight were *P. argentipes* females and five were *P. papatasi* females (Table 4).

**Table 4:** *Phlebotomus argentipes* and other sandflies collected for two consecutive nights of the entomological survey

Village, district	Altitude (m)	*P. argentipes*	*P. papatasi*	*Sergentomyia* spp.	Total female sandflies
Sanodhaple, Kavre	1,084	8	0	2	10
Sanonamja, Rolpa	1,351	0	0	0	0
Tebang, Rolpa	1,611	3	0	0	3
Palang, Dolpa	1,832	84	0	0	84
Sahartara, Dolpa	4,450	4	0	0	4
Tallokalkhe Humla	1,813	120	12	0	132
Tumcha, Humla	1,465	23	1	0	24
Madana, Humla	3,334	0	0	0	0
Firudi, Darchula	3,904	2	6	0	8
Khuna, Darchula	1,648	15	20	0	35
Lethya, Darchula	1,413	8	5	0	13

PCR testing for the kDNA of *L. donovani* in sandflies showed that none of the sandflies were positive.

### Assessment of knowledge, attitude, and practice and treatment-seeking behavior of communities in new VL foci.

The investigators together with local health facilities and the national program conducted awareness sessions on VL with the inhabitants of the investigated villages, where 17 from Kavre, 39 from Humla, 51 from Darchula, 35 from Dolpa, and 31 from Rolpa participated. A total of 182 household heads from the five districts were interviewed at baseline on their knowledge, attitude, and practices towards kala-azar, and 40 participated in the interviews at follow-up 6 months later. In comparison with baseline, 100% at follow-up mentioned fever as a symptom of kala-azar and that kala-azar transmitted by sandflies is a curable disease. Among the 52 participants who responded on protective measures from sandfly bites, 36.5% mentioned bednet use, 28.8% mentioned insecticide spray, and 11.5% mentioned use of repellents. At the 6-month follow-up interview, 67.5% mentioned insecticide spray, 20% mentioned bednet use, and 12.5% mentioned use of repellents as measures for protection from sandfly bites. Use of bednets had decreased at follow-up as this interview was conducted in winter. In the interviews at both baseline and after 6 months, 97.8% of the respondents at baseline and 100% at follow-up mentioned that they seek care from the doctor in case of illness. The most important factor for the choice of the health care provider and place was the condition of the sick person (Table 5).

**Table 5:** People’s knowledge of kala-azar and awareness of signs, symptoms, and transmission

Particulars	Baseline number (%) (*N* = 182)	Number (%) at 6 months (*N* = 40)
Had heard about disease	52 (28.6)	40 (100.0)
Kala-azar as a serious health problem	41 (78.8)	40 (100.0)
Knowledge of symptoms of kala-azar	*N* = 52	
Fever	28 (53.8)	40 (100.0)
Chills and rigor	2 (3.8)	0
Enlarged spleen	1 (1.9)	0
Enlarged liver	0 (0.0)	0
Skin blackening	1 (1.9)	0
Don’t know	20 (38.5)	0
Kala-azar as a curable disease	*N* = 52	
Yes	44 (84.6)	40 (100.0)
No	0	0
Don’t know	8 (15.4)	0
Sandfly bite causes kala-azar transmission	27 (51.9)	40 (100.0)
Protection measures	*N* = 52	
Use repellants	6 (11.5)	5 (12.5)
Insecticide spray	15 (28.8)	27 (67.5)
Use bednets	19 (36.5)	8 (20.0)
Bednets in the house	*N* = 182	
Yes	92 (50.5)	20 (50.0)
No	90 (49.5)	20 (50.0)
Respondent using bednets	*N* = 92	
Yes	82 (89.1)	19 (47.5)
No	10 (10.9)	21 (52.5)
Consultation person in case of illness	*N* = 182	
Consult doctor	178 (97.8)	40 (100.0)
Home treatment	1 (0.5)	0
Consult traditional healer	3 (1.6)	0
Factors affecting care-seeking	*N* = 182	
Condition of family member	178 (97.8)	40 (100.0)
Cost of seeking care	4 (2.2)	0

## DISCUSSION

We performed epidemiological, serological, and entomological investigations of new VL foci in villages from five districts reporting kala-azar cases within 3 years, from 2019 to 2021. We found that new focal villages of two districts, Dolpa and Kavre, had serological evidence of VL during the time of investigation. Entomological investigation showed that all new focal villages except Sanonamja in Rolpa and Madana in Humla, had female *P. argentipes* sandflies. There have been reports of VL cases from new focal hilly districts since 2010.^9^^–^^11^ Some of the studies performed epidemiological, serological, and entomological investigations and confirmed the indigenous transmission of VL in many new foci districts.^7^^,^^12^

There were very few symptomatic and asymptomatic cases of kala-azar in villages of the new focal districts. The serological investigation showed that only two new focal villages had seropositive cases. Although a few cases of splenomegaly and fever were detected, none of them were rK39 positive. Fever and splenomegaly are caused by many infectious and noninfectious inflammatory diseases, which require differential diagnosis using specific laboratory tests, including molecular tools. Unlike in the past, VL seroprevalence has been found to be significantly reduced even in districts of endemicity due to decreased transmission of *L. donovani* in Nepal.^13^

The epidemiological investigation of previous VL cases (2019 to 2021) showed that there might be indigenous transmission of VL in those villages because the majority of the patients had no travel history out of their village, but traders and visitors may have entered the village and brought the infection. Autochthonous cases of VL from hilly districts have been reported in Nepal since 2010.^14^ A study in the neighboring country of India found that VL is being increasingly diagnosed in residents of the high-altitude Garhwal region of the North Indian state of Uttarakhand due to migration of people carrying the parasite, which appears to have established a local transmission.^15^

In our study, we found that two of the previous patients with VL were already deceased at the time of investigation. Although we do not know the causes of death, comorbidity, relapse VL, and treatment toxicity could be the reasons for poor VL treatment outcomes.^16^ Therefore, follow-up of treated VL cases is very important.

In our study, mud houses with cracks and crevices, moist places around houses, animal sheds under the same roof of the house or a nearby house, and muddy places around the houses were observed in VL new foci districts. VL risk factors, including bamboo walls and cracks/water pooling inside and outside the house and around the animal shed, have been reported in other studies.^17^^,^^18^ Favorable sites for sandfly breeding should be targeted for sandfly control in these new foci areas.

Female and male *P. argentipes* sandflies were collected in most of the new focal villages at high altitudes included in the entomological investigation except for Madana village of Humla and Sanonamja village of Rolpa. The entomological study was conducted in mid-April in Humla and mid-September in Rolpa. The maximum temperature of these places was around 30°C in Madana and Sanonamja, whereas the minimum temperature was 17.6°C in Madana and 21°C in Sanonamja. *Phlebotomus argentipes* sandfly species have been incriminated as a vector for VL in the Indian subcontinent.^19^ These sandflies might have established local VL transmission in these villages. Other sandfly species like *P. papatasi* and *Sergentomyia* spp. were also found in significant numbers in some villages of new foci districts. Therefore, their potential role as a vector for VL transmission in Nepal needs to be investigated. However, in PCR testing for the kDNA of the *L. donovani* in sandflies, none of the sandflies was positive for parasite DNA. Previous studies conducted in Nepal that collected sandflies from Terai have shown that the natural rates of infection of *Leishmania* parasites in *P. argentipes* were less than 1%^20^ and 6.7%.^21^ Similarly, in Brazil, this rate was 1.56% in *Lutzomyia longipalpis*^22^ and 0.9% in phlebotomine sandflies.^23^ However, a study conducted in India revealed a high rate of infection of *Leishmania* in *P. argentipes* of up to 17.37%, but there was no correlation between the presence of infected sandflies and the occurrence of clinical VL.^24^

The abundance of VL vectors at the high altitudes under investigation revealed that ecological and climatic changes in these regions could be some of the factors associated with the shifting of VL to these new foci. The recent climate change might have already affected pathogen-vector-host systems in high-altitude regions of the tropics.^25^ A study in Pakistan found that the species abundance of sandflies at various elevations signify that these sandflies can survive in different environments at differing altitudes.^26^

People in new foci districts are not aware of kala-azar, its symptoms, the vector for transmission, or protective measures against sandfly bites. The investigators along with local health facilities and the national program conducted awareness sessions for the villagers, and these sessions had significantly improved their knowledge and practice in seeking care from clinicians in case of illness. Therefore, these educational interventions should be conducted extensively in new foci of VL to increase early care-seeking behavior in cases of VL transmission and to promote the practice of protective measures against sandfly bites. Although we have not explored delay times in seeking care in these new foci districts, another study revealed that the length of time that patients delay in seeking care for VL is significant in Nepal.^27^

In conclusion, our data point to an autochthonous transmission of VL in several new foci. This is a threat to the elimination efforts, as herd immunity is low in these areas and VL epidemics may occur in the future. Visceral leishmaniasis elimination activities should be implemented by the national program in these districts.
